# Targeting the Innate Immune Response to Improve Cardiac Graft Recovery after Heart Transplantation: Implications for the Donation after Cardiac Death

**DOI:** 10.3390/ijms17060958

**Published:** 2016-06-17

**Authors:** Stefano Toldo, Mohammed Quader, Fadi N. Salloum, Eleonora Mezzaroma, Antonio Abbate

**Affiliations:** 1 VCU Pauley Heart Center and Department of Internal Medicine, Virginia Commonwealth University, Richmond, VA 23298, USA; Fadi.Salloum@vcuhealth.org (F.N.S.); Antonio.Abbate@vcuhealth.org (A.A.); 2 Department of Cardiothoracic Surgery, Virginia Commonwealth University, Richmond, VA 23298, USA; Mohammed.Quader@vcuhealth.org; 3 VCU Johnson Research Center, Virginia Commonwealth University, Richmond, VA 23298, USA; emezzaroma@vcu.edu; 4 Department of Pharmacotherapy and Outcome Sciences, Virginia Commonwealth University, Richmond, VA 23298, USA

**Keywords:** heart transplantation, graft failure, rejection, donation after brain death (DBD), donation after cardiac death (DCD), inflammasome, Toll-like receptors, innate immune response, ischemia-reperfusion injury

## Abstract

Heart transplantation (HTx) is the ultimate treatment for end-stage heart failure. The number of patients on waiting lists for heart transplants, however, is much higher than the number of available organs. The shortage of donor hearts is a serious concern since the population affected by heart failure is constantly increasing. Furthermore, the long-term success of HTx poses some challenges despite the improvement in the management of the short-term complications and in the methods to limit graft rejection. Myocardial injury occurs during transplantation. Injury initiated in the donor as result of brain or cardiac death is exacerbated by organ procurement and storage, and is ultimately amplified by reperfusion injury at the time of transplantation. The innate immune system is a mechanism of first-line defense against pathogens and cell injury. Innate immunity is activated during myocardial injury and produces deleterious effects on the heart structure and function. Here, we briefly discuss the role of the innate immunity in the initiation of myocardial injury, with particular focus on the Toll-like receptors and inflammasome, and how to potentially expand the donor population by targeting the innate immune response.

## 1. Introduction

Heart failure (HF) affects more than 6 million people in the United States, and of those, approximately 10% suffer from advanced HF, requiring evaluation for mechanical circulatory support or heart transplantation (HTx) [1,2]. As of today, heart transplant is the only cure for end-stage HF [1]. As we get closer to the fiftieth anniversary of the first successful cardiac transplantation in 1967, heart transplantation has become a standard of care for patients with advanced heart failure [3]. After a steady rise in the number of heart transplants per year in the US, the number has now reached a plateau due to the limited number of available heart donors [3]. Meanwhile, the number of patients with advanced HF continues to increase tremendously [4]. In fact, the improvement in the treatment of patients with cardiovascular diseases and the progress in the management of ischemic and non-ischemic cardiomyopathies have reduced the overall mortality due to cardiovascular diseases at the expense of increasing the incidence and prevalence of HF [5,6,7]. 

The availability of an acceptable donor heart became the limiting factor for heart transplantation almost two decades ago [3,8]. This shortage of heart donors created a strong imbalance between the number of available hearts and the needs of the recipients, thus increasing the number of patients on the waiting list and prolonging the time to transplantation [3,8]. 

The International Society of Heart and Lung Transplantation (ISHLT) registry reported a total of 4196 adult and pediatric heart transplants performed in 2012 worldwide [3]. The majority of donor hearts came from subjects suffering from brain death after traumatic brain injury [3]. The 1- and 5-year survival for heart transplant recipients was 81% and 69%, respectively, with a median survival of 11 years [3]. The median survival was 14 years in recipients who were alive at 1 year after heart transplantation [3]. 

While these numbers are encouraging, they prompt consideration regarding the approximate 1 in 5 subjects who die within 1 year of the transplant, and the 1 in 3 who die within 5 years [3]. Improving our understanding of the pathophysiological events occurring during donor heart procurement, storage, and transportation, and the mechanisms of acute and chronic graft dysfunction resulting from the initial injury and from immunologic rejection, may lead to further improvements in short- and long-term survival. Moreover, while heart transplantation is now virtually limited to brain-dead donors (beating heart), it is conceivable that with the appropriate supportive therapies, heart transplantation from donation after cardiac death donors (fibrillated and arrested hearts) may become a reality [9]. This review will explore the pathophysiologic mechanisms of graft dysfunction after heart transplantation, as well as the opportunities and challenges of performing heart transplantation from donation after cardiac death donors (DCD). To facilitate the identification of the acronyms, in Table 1 we report the abbreviations used in this review in alphabetical order. We will focus especially on the role of the innate immune mechanisms, which serve as the body’s first-line response to not only pathogens but also to sterile insults like cellular stress or injury [10]. This response is stereotyped and is well conserved between different organs and organisms. For this reason, we will describe and discuss the evidence collected in the in preclinical and clinical data on the heart transplantation while also looking at other organs [10]. The optimization of heart transplantation procedures that would also allow heart transplantation from DCD donors has the potential to dramatically increase the number and the success rate of heart transplants [9]. With this goal in mind, we will describe the potential contribution of the innate immune response during every phase of the organ transplantation, from the identification of the donor to the post-transplantation course in the recipient.

## 2. Types of Donors

**Figure 1:**
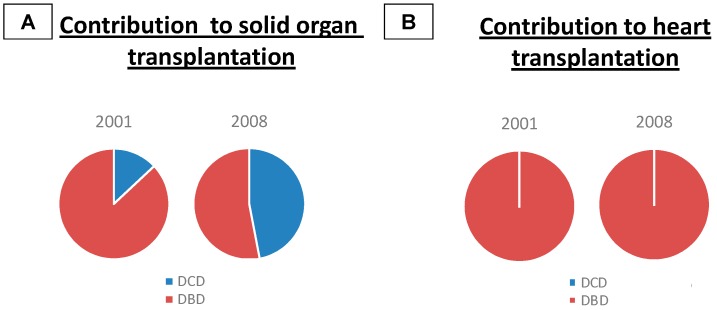
Contribution to solid organ transplantation divided per donor type. (**A**) The graph represents the relative percentage of organs derived from death donors in the years 2001 and 2008; (**B**) The source of hearts utilized in heart transplantation has not changed for the past two decades, deriving entirely from DBD. Abbreviations: DBD = donor after brain death; DCD = donor after cardiac death. Modified from [11].

The availability of donor organs is clearly the limiting factor in all solid organ transplantation, and the heart is no exception. The donation after brain death (DBD) represents virtually the entire cohort of heart donors (Figure 1) [3,8,9]. Patients with traumatic brain injury represent the larger portion of donors and account for 45% of the total of DBD, followed by massive strokes and anoxic brain damage [3,8]. 

Over the past years, with an attempt to increase the number of donors, “extended criteria” have been introduced in the HTx practice [12,13,14]. Typically, “extended criteria” refers to organ procurement from older donors, or from donors with clinical co-morbidities (*i.e.*, hypertension and/or diabetes) [3,15,16,17]. 

Another source of extended donor organs comes from patients who have been resuscitated after cardiopulmonary arrest [18]. The heart procurement from these donors, however, is suboptimal, and is hindered by the underlying cardiac dysfunction derived from the cardiac arrest [18]. From 2000 to 2013, the percentage of heart donors after resuscitated cardiac arrest used in the totality of HTx has doubled, though it still represents a very small minority of advanced HF cases [19]. Careful selection of these donors has shown safety and outcomes similar to the standard DBD donors, at least in the first years [20]. 

### Heart Transplantation from Donors after Cardiac Death 

As stated above, the rate of HTx has reached a plateau while the number of other solid organ transplantations (*i.e.*, kidney) has increased significantly over the years, primarily by utilizing organs from donors after cardiac death (DCD) [11,21]. 

In a study from the New England Outpatient Procurement Organization, DBD donor contribution of solid organs for transplantation went from 87% to 53% between 2001 and 2008, while DCD donor contribution increased from 13% to 47% (Figure 1) [22]. The use of DCD donors not only increases the available organs for transplantation, but has also had a significant impact on the overall wait times for transplantation and on the mortality of patients awaiting transplantation [23]. However, this has led to no changes in HTx, which remained a DBD procedure (Figure 1). 

HTx from DCD is characterized by inherent challenges related to cardiac death and organ harvesting [24,25,26,27]. DCD entails several steps during which the myocardium is subjected to the deleterious effects of hypoxia and ischemia, which certainly injure the heart [9,25]. Generally, DCD is considered when the donor is found inevitably destined to die, but is not brain dead. The donor is therefore followed closely by the transplant team, and organ explant occurs only after cardiac arrest. In most instances, termination of the ventilator support, according to the wishes of the donor’s next-of-kin, is performed in the operating room area. After anoxia ensues, cardiac arrest is inevitable. Once pronounced dead, the organs are harvested.

**Figure 2:**
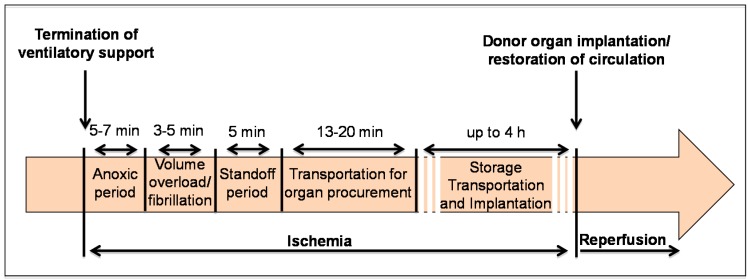
Steps and timing of the donation after cardiac death protocol (DCD). This timeline is based on the standard DCD organ procurement protocol and incorporates the timing for storage, transportation and implantation that are applied to the Donation after Brain Death heart. The DCD heart becomes ischemic starting from the termination of ventilator support and ensuing anoxia. Initial ischemia occurs in the donor (warm ischemia) and lasts approximately between 25 and 35 min. The storage and transportation may increase the time of ischemia (cold ischemia during storage and preservation) for an additional 4 h. With the heart transplantation and the restoration of the circulation, the donor’s heart is reperfused within the recipient’s circulation.

The main steps of the DCD process are described below and a graphic timeline with the average time intervals for each step is reported in Figure 2. 

The increasing availability of DCD donors represents a unique opportunity to increase the number of hearts available for transplantation [23]. A recent study showed that when the DCD donors were matched for selection criteria with DBD heart donors, the authors noted a 15% match [28]. This would have resulted in a 17% increase in the heart donation in the authors’ Organ Procurement Organization in Wisconsin [28]. A similar study was done in Belgium where a 15% increase in overall HTx activity was noted if the DCD donor hearts could be utilized for transplantation [29]. In the same study, the impact of this potential 15% increase in available hearts for transplantation would have decreased the mortality on the transplantation waitlist by 40% [29]. 

Mechanical circulatory support devices, such as left ventricular assisting devices (LVADs), are often used to temporarily assist patients waiting for heart transplantation [30]. LVADs have safely extended the wait times for patients, but the number of patients who ultimately receive a transplant still represents only a small fraction [31,32].

The economic impact of heart transplantation from DCD donors is also quite large [32]. Many patients on transplant waitlists are supported with intravenous inotrope treatments and/or ventricular assist devices, adding to the total cost of care [33]. 

In order to consider heart transplantation after DCD, it is necessary to characterize the type and degree of cardiac injury occurring during the DCD process and to develop the strategies needed to protect, preserve, and prepare the hearts for re-implantation [24,25]. A recent report showed that perfused and rehabilitated human DCD hearts can be successfully transplanted in donors with promising results [34].

## 3. Myocardial Injury during Organ Procurement

Donation from living donors is feasible in many forms of solid organ transplantation, but for obvious reasons, it is not an option for HTx [3]. 

The success of organ transplantation from DBD is inferior compared to the transplantation of organs from living donors, as reported by some studies that suggest brain damage somehow impairs the function of the transplanted organ [35,36,37,38]. 

Retrospective studies have showed that the time of ischemia inversely correlates with the success of organ transplantation [39]. In DBD, the time of warm ischemia is minimal, and cold ischemia is the major contributor to myocardial damage [40,41,42]. Warm ischemia is referred to as the lack of blood flow and tissue oxygenation that occurs at normal body temperature [39]. The cell metabolism stays high at normal body temperature. The occurrence of ischemia leads to faster consumption of energy and intracellular substrates, and the accumulation of metabolic byproducts [39]. The most common form of warm ischemia that occurs in the heart is due to coronary artery disease [43]. The reduction of the lumen of the coronary artery (due to growth of an atherosclerotic plaque or to plaque rupture) causes a regional poor tissue perfusion [43,44]. In contrast to warm ischemia, cold ischemia happens at a very low cellular metabolic rate. Due to the low temperature reached during organ storage, cellular damage is reduced [42,45,46,47,48]. In heart transplantation, the maximum tolerated time of cold ischemia is estimated at 4 h, which is considerably shorter than other organs with a lower metabolic rate (e.g., liver and kidney) [49]. Cold ischemia is practically inevitable due to the need of transporting the donor organ [41,42]. Storage solutions have been optimized to preserve organs during transportation [50]. Heart preservation goes through two processes: the cessation of heartbeat, using a cold cardioplegic solution, and the cold storage [41]. Cessation of heartbeat reduces energy expenditure by >90%. For the flushing and storage of an organ to be transplanted, there are several solutions, developed to preserve or protect the ischemic organ, and often optimized for each organ [41]. In heart transplantation, hyperkalemia is one of the requirements for the cardioplegic solution, and the St. Thomas™ solution is the most commonly used solution [41]. Blood-based solutions are also used for this type of intervention, by mixing the donor’s blood with a cardioplegic solution [41]. The storage can then be carried out in two different ways, either by using static cold storage (SCS) or by hypothermic machine perfusion (HMP), which is capable of giving metabolic support [41]. In preclinical studies, the HMP is found superior to the SCS in preserving the DCD hearts because of the increased metabolic support [50,51]. 

A recent randomized study conducted in DBD kidney donors shows that kidneys from donors kept in mild hypothermia (34.0–35.0 °C) *vs.* normothermia (36.5–37.5 °C) after brain death had significantly decreased rates of delayed graft dysfunction [52]. 

Ischemia represents one of the challenges of the organ procurement and storage protocols [41,44,46,47]. Ischemia hinders ATP production and the cellular homeostasis, leading to uncontrolled fluid re-distribution and cellular edema [44,53,54]. Simultaneously, there is an increase in the extracellular pH and fluid stasis in the capillaries. This produces capillary damage and decreases the perfusion capacity of the capillaries [55,56]. In addition, reperfusion injury occurs at time of the actual transplantation [54,55,56]. Reperfusion injury is intrinsic to the reperfusion and reoxygenation process [57]. The reestablishment of the physiological amount of oxygen following a sustained period of ischemia can be a source of reactive oxygen species (ROS) [58]. ROS are important mediators of cellular signaling but also of injury [59]. A surge in ROS occurs when mitochondria rendered dysfunctional during ischemia are re-exposed to oxygen, and result in a production of ROS through NADPH oxidases and xanthine oxidase. An excessive production of ROS damages DNA, intracellular proteins, and enzymes, potentially leading to cell death [58,59]. 

Unlike other solid organs, however, the heart has a high metabolic need that makes the heart particularly sensitive to ischemia and to reperfusion injury. The use of DBD characterized a giant leap forward in the heart transplant field, leading to surgical success and to a functional transplanted heart. These considerations have historically prevented the use of DCD hearts for transplantation due to the fear of early graft failure [9]. Warm ischemia during the DCD protocol (anoxia-induced cardio-respiratory death) results in significant myocardial damage that is proportional to the duration of time between the withdrawal of support and cardiac death [9]. Moreover, the heart undergoes a second wave of injury upon implantation and restoration of blood flow (reperfusion injury), primarily due to oxidative stress and inflammation [55,56,57,58,59,60]. The lack of oxygen during anoxia in the DCD protocol induces a large increase (50-fold) in plasma catecholamine levels, further inducing cardiomyocyte injury [61]. The warm fibrillating heart continues to expend increasing amounts of energy and thus decreases ATP and increases low-energy phosphates [62]. In this phase, the stasis of blood induces endothelial damage. This phase is referred to as ‘warm ischemia’, leading to the moment of heart procurement [62]. During organ explant (procurement), the DCD heart is then exposed to ‘cold ischemia’ as it occurs during DBD heart transplantation. The ‘warm ischemia’ prior to organ explant, however, likely serves as a primer for further injury during ‘cold ischemia’, exacerbating the effects of ischemia and reperfusion injury, and making the injury more severe.

Neurohormonal activation during DCD heart transplantation contributes to further damage before the cardiocirculatory arrest [63,64]. Reperfusion following transplantation floods the donor heart with ROS and inflammatory mediators that have accumulated (in both donor and recipient tissue) during the ischemic periods [55,56,57,58,59]. The introduction of ROS creates a surge of tissue injury, leading to cell damage, cell death, and a second wave of inflammation [57]. 

**Figure 3:**
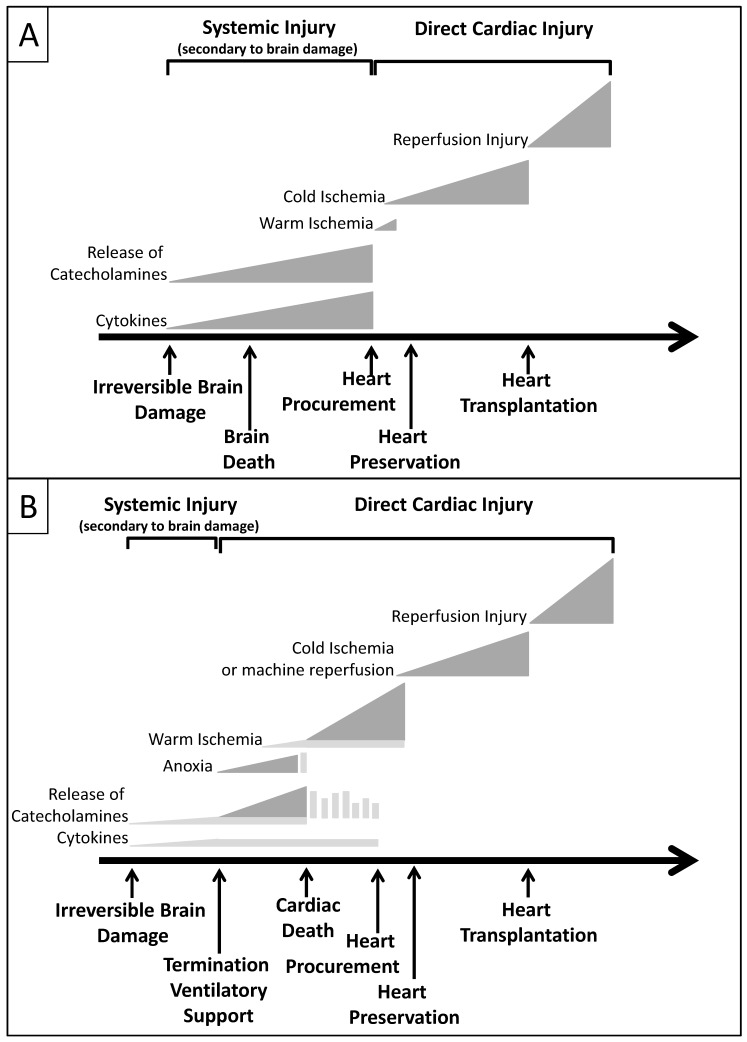
Different mechanisms of injury to the donor heart before and after procurement, storage, and transplantation, in the DBD and the DCD hearts. The DBD heart (**A**) is exposed to a systemic injury, driven by the damaged brain that increases catecholamines and circulating cytokines (point further discussed in the next section). Heart procurement initiates a local and direct injury to the myocardium due to warm and cold ischemia. Impact of warm ischemia is considered minimal in the DBD heart. Reperfusion due to transplantation and resuscitation further increases the damage. In the DCD heart (**B**), anoxia and the long period of warm ischemia increase the heart injury. Based on the literature, machine perfusion is an alternative to cold ischemia for organ preservation and transportation of DCD hearts [34].

From the identification of the donor to the transplantation, the donor heart is exposed to several types of injury (Figure 3). Each of these steps is a trigger for the inflammatory response (see next section) affecting cardiac function. 

## 4. Innate Immune Response during Organ Procurement

The explanted heart from DCD is therefore injured before procurement by severe hypoxia and the surge of plasma catecholamines. Once transplanted in the recipient, the explanted DCD heart is exposed to the ischemia-reperfusion injury, leading to cardiac dysfunction. Once the heart is grafted and beating, the transplant faces the additional hurdles of immune-mediated rejection [3,8]. 

With the progress of donor-recipients matching programs and immune-suppressive therapies, acute antigen-mediated rejection has become less prominent compared to the past [65]. However, despite this achievement, the inflammatory response still plays a central role in determining the success of organ transplantation [3,8]. 

Pre-clinical and clinical studies have highlighted that the innate immune response, which is an antigen-independent inflammatory mechanism, contributes to organ dysfunction and enhances acute allograft rejection [66,67]. The molecular pathways activated by the cellular response to tissue damage initiate a cascade defined as sterile inflammatory response. The debris and byproducts of a damaged cell (*i.e.*, ATP, adenosine, hydrogen and potassium ions, and the release of intracellular alarmins) are referred to as Damage Associated Molecular Patterns (DAMPs) and serve as the initial triggers for the sterile inflammatory response [68,69]. DAMPs represent a heterogeneous group of often structurally unrelated molecules, and as such they bind to a wide series of Pattern Recognition Receptors (PRRs). With DAMPs, PPRs are part of the innate immune system. Together they lead to the coordinated activation of inflammatory pathways in the resident cells, as well as to the recruitment of leukocytes to the site of injury [66,68,69]. PPRs like the Toll-like receptors (TLRs) and the NOD-like receptors (NLRs) are among the best characterized, due to their central and conserved role in response to tissue and cellular injury [68,69]. The response to ischemic injury to the heart is a classic example of sterile inflammation [68]. Identifying the DAMPs and the PRRs associated with the activation of sterile inflammation during heart transplant may help to define better strategies to blunt the negative effects of this pathway on organ recovery, function and longevity following transplantation.

### 4.1. The Inflammasome

The inflammasomes are the first-line element of sterile inflammation [69]. They act as guardians, bridging the sensing of cell damage to the activation of the inflammatory response [70,71]. There are several inflammasomes, classified based on the sensory component. The sensory part of the inflammasome in the heart is a cytosolic PRR named NLRP3 (NOD-like receptors [NLR] containing a Pyrin Domain 3) [70,71]. This is a tripartite large protein with a central NOD domain, a receptor domain at the C-terminal (series of leucine-rich repeats –LRRs–) and an N-terminal PYRIN domain (PYD) and is activated in response to the activity of several DAMPs. The active NLRP3 binds the adaptor protein ASC (apoptosis speck-like protein containing a caspase recruiting domain-CARD-), which in turn interacts with the CARD of caspase-1 [7]. 

**Figure 4:**
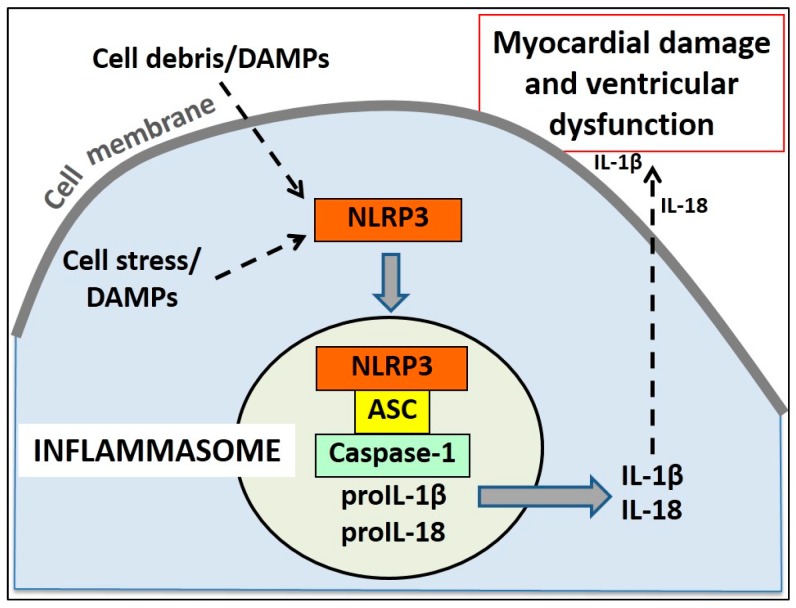
Schematic representation of the signaling pathway of the NOD-like receptors (NLR) containing a Pyrin Domain 3 (NLRP3) inflammasome following myocardial ischemic injury. Extracellular debris and intracellular stress signals activate the “danger sensor” NLRP3. NLRP3 recruits the adaptor protein ASC and the effector enzyme caspase-1. Caspase-1 converts the pro-forms of IL-1β and IL-18 into the biologically active forms, which are released into the interstitial space. In severe cases, the persistent inflammasome activity induces cell death. The release of active IL-1β and IL-18 induces further myocardial damage and ventricular dysfunction.

Recruitment of caspase-1 into the inflammasome favors its autocatalytic activation, a step necessary for the processing of the pro-inflammatory cytokines of the IL-1 family, IL-1β and IL-18 [70,71]. The NLRP3 inflammasome activation leads to the amplification and progression of the inflammatory response through the release of inflammatory cytokines. In the heart, this can be particularly detrimental for the cardiomyocyte function, impairing contraction and in severe cases inducing pyroptosis, a caspase-1-dependent cell death (Figure 4) [70,71,72,73]. In the heart, the NLRP3 inflammasome formation activity correlates with the intensity of myocardial damage in animal models of ischemic and non-ischemic cardiomyopathy [69,74,75,76,77,78,79]. 

### 4.2. Inflammatory Injury in the DBD Heart

The deleterious effects of the procurement process of the donor heart are not well-defined. In DBD, the cardiovascular system has impaired sympathetic and parasympathetic autonomic control, causing changes in the myocardial perfusion and cardiac output, and increasing myocardial levels of adenosine, lactate and catecholamine [80,81]. These signals can act as triggers of innate immunity [69]. The rise in catecholamine leads to calcium overload and compromises cardiomyocyte contraction/relaxation [63]. A preclinical study has also demonstrated myocardial neutrophil infiltration in a DBD model 48 h following transplantation [82]. The same study showed that the neutrophils were of donor’s origin. Other animal studies have shown that brain death induces myocardial deposition of the complement C3a, and that inhibiting the complement cascade reduces the inflammatory response developed in the heart graft and increases graft survival [83,84]. The same response was observed in a model of kidney transplantation in the mouse [85,86]. Similarly, the DBD kidney increased the expression of several cytokines in mice. Therefore, brain death itself induces myocardial damage and an inflammatory response [87]. However, *ex vivo* experiments using human blood have shown contrasting effects of catecholamines on the release of IL-1β. A physiological rise of catecholamines induces IL-1β [88]. However, catecholamines have an inhibitory effect when LPS is used as a stimulus for IL-1β [89]. This discrepancy may be explained by increased utilization of ATP induced by catecholamines. This would reduce the monocytes’ LPS-mediated IL-1β release triggered by the release of ATP in the extracellular space [72].

Recent preclinical studies have shown that in DBD donors the levels of IL-1β are increased in the heart tissue and in the plasma [90,91,92]. Additionally, in non-human primates, treatment of the brain death donors with the IL-1 blocker, recombinant IL-1Ra, increases the post-transplantation survival of the pancreatic beta islets to levels comparable with the islets collected from living donors [93]. IL-1 is one of the key regulators of the innate immune response, which is up-regulated by ischemia and reperfusion injury. Data on the production of IL-1β or of the protective effects of IL-1Ra in the DCD heart are currently lacking. 

### 4.3. Inflammation during Cold Ischemia 

**Figure 5:**
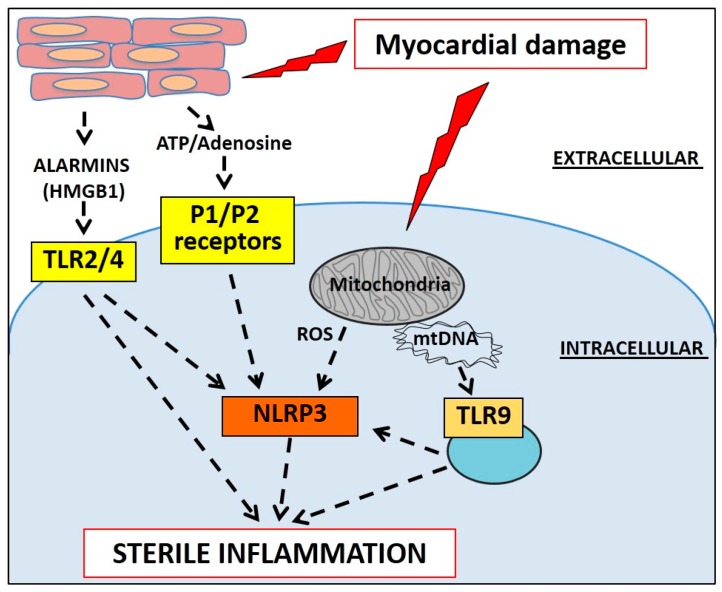
Myocardial injury activates the innate immune response. Alarmins and purines released by injured cells activate the Toll-like receptors (TLRs) and the P1 and P2 purinergic receptors. Damaged mitochondria produce reactive oxygen species (ROS), activating the NLRP3 receptor, and expose mitochondrial DNA to TLR9 in intracellular vesicles. TLR signaling converges also on the NLRP3 inflammasome signaling. All together, these pathways contribute to the activation of the sterile inflammatory response.

The cold storage phase, characterized by cold ischemia, can also itself have pro-inflammatory effects [90]. In animal models of heterotopic heart transplantation, the cold storage phase leads to the release of alarmins (*i.e.*, HMGB1 and IL-17A), engaging more neutrophils in the transplanted heart [91]. Alarmins such as HMGB1 act as DAMP binding to the respective PRRs, *i.e.*, Toll-like receptor-4 (TLR-4) for HMGB1 (Figure 5) [91]. Myocardial damage increases with time of cold ischemia and the use of a mitochondria-targeted anti-oxidant appears to reduce the injury and production of pro-inflammatory cytokines [92]. Oxidative damage generated by dysfunctional mitochondria is a source of various DAMPs [93,94]. During ischemia, damaged mitochondria generate ROS, oxidized mitochondrial DNA and induce lysosomal dysfunction, activating PRRs such as the NOD-like receptor protein-3 (NLRP3) and TLR-9 [71,95]. Although these studies show that ischemia represents a trigger for inflammation, human studies are currently lacking. The analysis of kidneys from human patients exposed to SCS or HMP, however, revealed that cold ischemia has a pro-inflammatory activity that is reduced with the use of HMP [96]. Whether this could be reproducible in human hearts is yet to be determined. 

The exposure of DCD hearts to a relevant time of warm ischemia, and to the catecholamine storm that precedes cardiac arrest, functions as a primer for inflammation [61]. Animal models of acute myocardial infarction have indeed shown that ischemia is a potent trigger of inflammation [69,97,98].

## 5. Primary Graft Dysfunction after Transplantation

**Figure 6:**
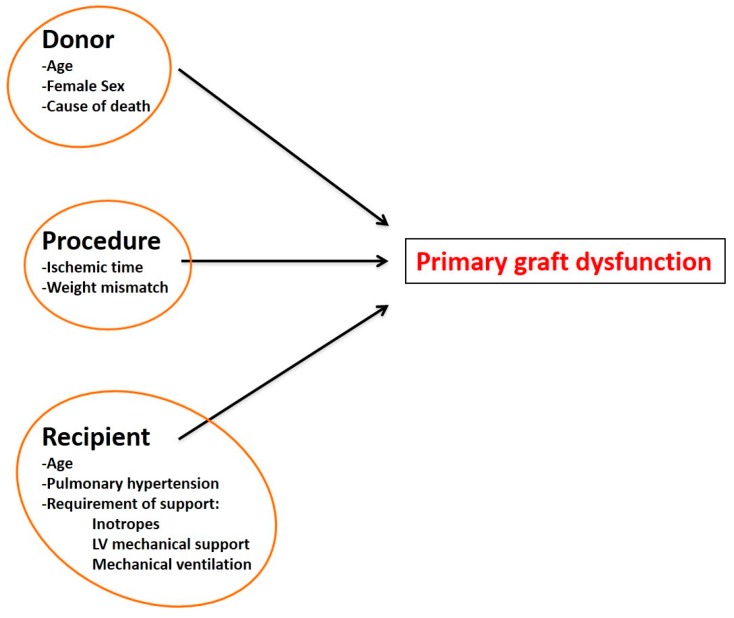
Risk factors for primary graft dysfunction. Donor characteristics, variables linked to the transplantation procedure, the characteristics and the post-transplant care of the recipient are among the risk factors associated with primary graft dysfunction (PGD).

Primary graft dysfunction (PGD) refers to reduced heart function in the early post-operative period, due to left and/or right ventricular impairment requiring inotropes treatment and/or mechanical circulatory assist devices [63,99,100]. PDG occurs in the first 24 h following the transplant and its occurrence is associated with poor outcomes [63,100]. PDG requires inotropes administration and, in the most severe cases, mechanical and ventilatory support [100]. The causes are not clear, but likely include the increase in catecholamines plasma levels due to brain death, the ischemia during procurement and storage (then followed by reperfusion injury), and the need for high-dose inotropes or vasopressors to resuscitate the heart leading to β-Adrenergic Receptor (β-AR) desensitization and stunning [63,99,100]. Several risk factors have been identified and are listed in Figure 6. 

Pro-inflammatory cytokines (e.g., IL-1, IL-18 and TNF-α) have cardiodepressant activity and thus may contribute to PGD [73,101]. High inflammatory activity is seen in several of the conditions identified as risk factors for PDG. Brain death, catecholamines, and older age of the donor, along with the ischemic damage during the procurement and storage, expose the heart to pro-inflammatory signaling before transplant. Furthermore, the preexisting heart failure condition of the recipient is associated with high levels of systemic pro-inflammatory cytokines. The use of inotropes in the recipient may further contribute to heighten the cytokine levels and promote adverse cardiodepressant pathways [55,56,57,58,59,82,83,84,85,86,87,100,101].

## 6. Immune Response Leading to Acute or Late Rejection

Graft failure is the leading cause of death for all heart transplant recipients, followed by infection and multiple organ failure [3]. 

### 6.1. Hyperacute and Acute Rejection

Hyperacute rejection is an antibody-mediated rejection due to the presence of pre-formed antibodies in the recipient [102]. Antibody detection assays are performed before HTx to prevent hyperacute rejection [103,104]. Acute rejections account for approximately 10% of deaths in the first 3 years, but acute and chronic immune injuries are likely important contributors to graft failure [3]. This occurs despite progress made to match donors and recipients, improved organ preservation and storage, as well as progress toward increased knowledge on the use of immunosuppressive therapies in the recipient. Immunosuppression is commonly achieved with a triple drug regimen, with corticosteroid, calcineurin inhibitors, plus an anti-proliferative agent [3]. 

Two different types of acute graft rejection can occur: acute cellular rejection (ACR) and acute antibody-mediated rejection (AMR) [105]. Rejection has no definite symptoms and the sampling of endomyocardial biopsies is the gold-standard method for early diagnosis of allograft rejection [105]. ACR is initiated by T-lymphocytes and is characterized by the migration of lymphocytes and macrophages into the myocardium, with devastating effects on cardiomyocyte survival [105]. ACR is graded with a score from 0 to 3, using standard histopathology of the myocardial biopsies [105]. A grade 0 represents no signs of ACR, while grade 3 is severe cellular rejection. ACR develops more frequently than AMR [3,106]. The latter, is characterized by complement and B-lymphocyte activation, which produces antibodies against leukocytes and endothelial antigens of the donor organ [106]. Sometimes the donor organ already presents these antibodies before transplantation [107]. For this reason, diagnosis of AMR is done with immuno-staining against immunoglobulins, complement deposits or macrophages within capillaries, using peroxidase-based or fluorescence-based staining [107]. Although less frequent, AMR portends increased mortality, more pronounced myocardial damage, and more cardiovascular complications than ACR [106,107].

### 6.2. Late Rejection

AMR and ACR also increase the incidence of cardiac allograft vasculopathy (CAV), a type of coronary artery disease associated with late rejection, which increases the risk of graft failure and death [105,106]. CAV is associated with endothelial dysfunction, increased cytokine production, and the presence of lymphocytes and macrophages in the intima of the coronaries [106]. Although the pathogenesis of CAV is unknown, pre-existing conditions in the donor, like coronary artery disease, have shown a strong association with its development [106].

The improvement in our knowledge on the pathophysiological mechanisms of acute and chronic rejection, CAV, coupled with an increase in the number of heart donors and improved organ preservation during procurement, storage and transplantation may be the key to augmenting the numbers and success of future HTx.

## 7. Immune Response following Transplantation

### 7.1. Lymphocytic Response

T- and B-lymphocytes mediate the rejection mechanisms of ACR and AMR [105,106]. T-lymphocytes have the ability to recognize non-self major histocompatibility complex (MHC) molecules, through the activity of antigen presenting cells (APC) transiting in the graft [108]. This induces the T-cell transition into effectors, which makes them reactive against the graft [108]. In addition, the expression of the adhesion molecule selectins and the MHC are increased in DBD organs, which may increase T-lymphocyte reactivity against the graft [87]. 

### 7.2. Innate Immunity and Myocardial Injury following Transplantation

**Figure 7:**
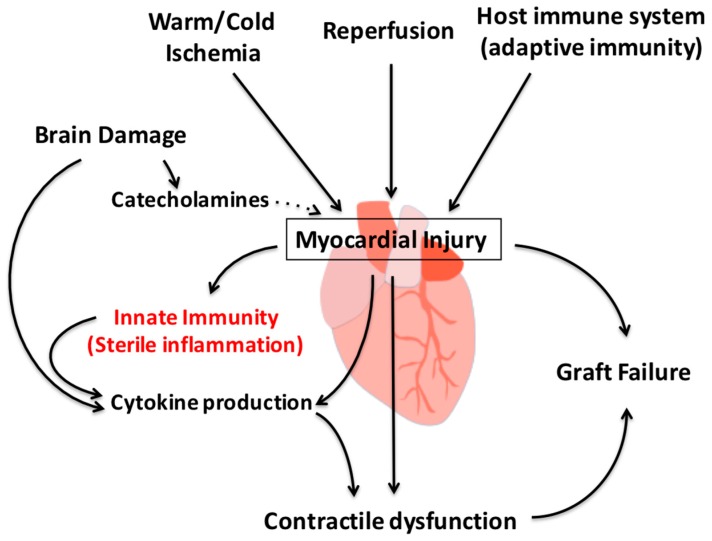
Myocardial injury triggers activation of the innate immunity. Ischemia, reperfusion injury and the host immune system induce injury to the heart, amplification of the inflammatory response, and production of pro-inflammatory cytokines. This phenomenon leads to further injury and contractile dysfunction, ultimately leading to graft failure.

Innate immunity is a mechanism activated by ischemia and reperfusion injury and considered an alternative to the adaptive, lymphocyte- and antigen-mediated immunity. This innate immune response, together with the systemic inflammation that follows brain injury or death and the adaptive immunity post-transplantation, induces myocardial injury and dysfunction (Figure 7). 

Innate immunity represents a stereotyped mechanism that has evolved to promptly respond to injury occurring as a consequence of invading microbiologic pathogens (infectious inflammation) or in response to non-infectious tissue injury (sterile inflammation) [68,69]. As such, innate immunity is activated following several types of injury or stress signaling and occurs at an organ or cellular level by the presence of DAMPs and through the activation of PRRs [66]. This signaling pathway is more complex, and involves several molecules and regulatory proteins [66]. The complexity is further increased since different players are activated at different stages of organ procurement and transplantation or in different ways according to the type of donor death (DBD *vs.* DCD). Identifying the central nodes in the sterile inflammatory response common to the ischemic injury, to DBD- and to the DCD-associated myocardial damage will help to develop *ad*-*hoc* strategies to reduce graft failure. 

## 8. The Innate Immune Response as a Potential Pharmacological Target

Several types of PRRs have been identified; some are involved exclusively in the pathogens associated molecular patterns (PAMPs), and others with dual function of recognizing PAMPs and DAMPs [66]. 

### 8.1. Interleukin-1α and Its Role as Alarmin

Interleukin-1α (IL-1α) is expressed on the endothelium of coronary arteries and increases T-cell adhesion to the endothelium [109]. IL-1α belongs to the IL-1 family of cytokines and is particularly relevant in the signal transduction of the innate immunity [73]. IL-1α is present as an active form in intact cells, and is either expressed on the membrane or released by damaged or dying (necrotic) cells, acting as an alarmin [73]. IL-1α shares similarities with IL-1β, which, unlike IL-1α, requires processing from pro-IL-1β to its mature form and is actively produced only during inflammation [73]. Therefore, IL-1α acts as a DAMP, while IL-1β represents a mediator and amplifier of the inflammatory response. The two forms of IL-1 bind to the same IL-1 receptor type I (IL-1RI), which induces the transduction of the intracellular signal with relevant consequences for the inflammatory response [73]. IL-1 signaling induces activation of nuclear factor-κB (NF-κB) and induces the expression and release of several “secondary” cytokines, with relevant consequences for the initiation, amplification and sustainment of the inflammatory response [73]. This pathway is reviewed in detail below. 

### 8.2. Targeting the Toll-Like Receptors (TLRs) Pathway

The TLR family of receptors is commonly involved in this type of dual recognition of PAMPs and DAMPs, and among other PRRs, TLRs are better characterized [110,111]. In mammals, there are 12 identified TLRs involved in the recognition of bacteria, fungal or viral products [108]. These are type-I integral membrane receptors, with leucine-reach repeats (LRRs) in the extracellular domain [110]. The TLRs share a similar structure and mechanism of activation, working as homo- or hetero-dimers [110]. Upon binding to their ligands, the extracellular domains of two TLRs get closer, leading to interaction of the C-terminal Toll/Interleukin-1 receptor (TIR) domain of the two TLRs [108]. The TIR domain is present also in the intracellular C-terminal of the IL-1RI receptor and other receptors of the IL-1 family [73,110]. The TIR domain transduces the signal by interacting with different intracellular protein adaptors (*i.e.*, MyD88, TIRAP, TRAP and TRIF) [69,110,111]. MyD88 (Myeloid Differentiation Factor 88) interacts with the TIR of almost all the TLRs, with the exception of TLR-3 [110]. Like IL-1RI signaling, the TLR signaling activates NF-κB, as well as the Mitogen Activated Protein Kinases (MAPK) signaling and members of the Interferon Regulated Transcription Factors (IRFs) [110]. All together, the TLRs recognize a wide spectrum of PAMPs and DAMPs [110]. A few examples of TLR agonists active in ischemia-reperfusion injury are alarmins (e.g., heart shock proteins, HMGB1), fragments derived from degradation of proteins of the extracellular matrix degradation (e.g., fibronectin, fibrinogen, hyaluronan, S100), and mitochondrial DNA [112]. HMGB1 is increased following traumatic brain injury, and thus may sensitize the organs to tissue injury or contribute to altered organ function [113]. 

Expression of TLR-2 and TLR-4 increases due to ischemia-reperfusion injury in kidney transplants and the donor TLR-4 mediates ischemia-reperfusion injury [114,115]. TLR-2 and TLR-4 are constitutively expressed in the heart and drive ischemic injury to the heart in animal models of myocardial ischemia-reperfusion injury [69,113]. HMGB1 is increased in mouse cardiac isograft due to ischemia-reperfusion [91]. In this model, TLR-4 deletion reduced acute cardiac injury [91].

TLR-2 is increased in human transplanted kidneys during acute rejection. Several experimental studies in the mouse support the role of TLRs in acute rejection [116]. Gene silencing of the TLRs intracellular adapters MyD88 and TRIF delays graft rejection in a mouse model of heterotopic heart transplant [117]. Experimental kidney transplant has also showed that TLR-2 and TLR-4, and the MyD88 and TRIF signaling, contribute to chronic graft dysfunction [118]. These data support the hypothesis that pharmacological blockade of TLR signaling has protective effects during all phases of heart transplant. 

### 8.3. Targeting the NLRP3 Inflammasome Pathway

The inflammasome has a key role in the early sensing, activation and amplification of inflammation in response to tissue injury. The activation of the inflammasome in the heart requires two independent processes, the priming and the triggering; one without the other is insufficient to induce cardiac dysfunction [119]. 

Ischemia triggers the activation of the NLRP3 inflammasome both *in vivo* and *in vitro* [75,120]. *In vivo*, purines seem to be important triggers of NLRP3 activation. Inhibition of the ATP-activated purinergic receptor P2X7R, the adenosine receptor AdoR2B, or their individual gene silencing are mechanisms sufficient to blunt caspase-1 activation and to induce cardioprotection [69,75,121]. Data obtained with cultured cardiomyocyte exposed to ischemia, on the other hand, suggested that the inflammasome activation is, at least in part, independent from extracellular ATP release, thus suggesting mitochondrial dysfunction as an independent trigger [69]. 

Data on the role of the inflammasome during organ procurement, storage, or reperfusion due to transplantation are currently lacking. A recent report showed that IL-18 blockade using IL-18 binding protein (IL-18BP) improved graft survival in a mouse model of syngeneic heterotopic heart transplantation [122]. IL-18BP expression of pro-inflammatory cytokines (including IL-1β), reduced cardiomyocyte necrosis and infiltration of CD4^+^ T-lymphocytes, macrophages and neutrophils. The inflammasome regulates acute graft *versus* host disease in an experimental model of hematopoietic cell transplantation [123]. In the same study, increased expression of caspase-1 and IL-1β was observed in tissue samples of patients presenting with graft *versus* host disease [123]. In the mouse, cardiac allografts present diffuse protein expression of the inflammasome component ASC and IL1β, suggesting activation of the inflammasome pathway [124]. A recent study also showed that cardiac biopsies collected to monitor tissue rejection were positive for ASC specks, indicating the presence of the inflammasome [125]. ASC positivity directly correlated with the severity of cellular rejection and early death caused by heart failure [125]. Interestingly, myocardial samples collected during organ procurement, in human DBD and DCD donors, showed that DCD hearts express more caspase-1 and NF-κB mRNA [126].

Inflammasome inhibitors to be used in clinical practice today are lacking [127]. A small molecule inhibitor derived from glyburide developed at the Virginia Commonwealth University (Richmond, VA, USA) has shown to reduce myocardial injury in animal models of ischemic and non-ischemic heart disease [128,129,130]. A novel inhibitor, also derived from glyburide, MCC950, developed in Ireland has been shown to inhibit NLRP3 *in vitro* and *in vivo*, but it has not been tested in animal models of cardiac diseases [131]. Targeted inhibition of the NLRP3 inflammasome prior to harvesting, during harvesting and transport, or after transplantation may prove useful in reducing myocardial injury, thus potentially preventing primary graft dysfunction and the secondary stimuli for the immune rejection.

Reduced NLRP3 activation can be reached by inhibiting the P2X7 receptor [75]. In fact, following experimental acute myocardial infarction, P2X7 inhibition blunts caspase-1 activity and reduces myocardial damage [75]. Similar results were observed with inhibition of the adenosine receptor AdorA2B [121]. P2X7- and AdoRA2B-targeted drugs are currently undergoing clinical development [132,133].

Additional strategies that reduced myocardial ischemia-reperfusion injury by inhibiting caspase-1 in the mouse were alpha-1 antitrypsin (AAT) or derived small peptides. Plasma-derived or human recombinant AAT, or genetically engineered small peptides designed to recapitulate the anti-inflammatory effects of the C-terminal peptide of AAT and other serine protease inhibitors (SP16, Serpin Pharma, Manassas, VA, USA), have shown to significantly reduce infarct size in myocardial ischemia-reperfusion injury in the mouse [134,135]. Na_2_S, a hydrogen sulfide donor, has also shown promising effects on ischemia-reperfusion injury and inflammasome inhibition [136].

NF-κB signaling, mediated by the TLR or other PRRs and/or cytokine/chemokine receptors, is also an important determinant of inflammasome activation [69]. NF-κB drives the transcription of the inflammasome components and cytokines [69]. Inhibition of NF-κB reduces myocardial ischemia-reperfusion injury and caspase-1 in mice, likely by inhibiting inflammasome priming [137]. 

Studies to characterize the functional role in injury, organ dysfunction and tissue rejection of the inflammasome in the heart, however, are lacking.

### 8.4. Interleukin-1 Blockade

IL-1β blockers are considered the standard of care for the clinical treatment of inflammasome-mediated diseases [138,139,140]. Anakinra is a recombinant form of IL-1Ra and blocks IL-1α and IL-1β signaling [73]. Rilanocept is a chimeric protein developed by conjugating the ectodomains of the IL-1RI and the IL-1R Accessory protein (IL-1RAp), which also neutralize both the forms of IL-1 [73]. A third blocker, canakinumab, is a blocking antibody that selectively blocks IL-1β [73]. Anakinra was shown to reduce graft *versus* host disease in mice [123]. The 3 blockers, as well as genetic manipulation of the IL-1RI pathway, exert cardioprotection in animal models of ischemic heart disease [141,142,143,144,145,146,147].

Anakinra is currently tested in clinical trials to evaluate its anti-inflammatory and cardioprotective effects in patients with acute myocardial infarction (AMI) and/or heart failure [148,149,150,151]. In a pilot clinical trial of patients with large AMI, anakinra blunted the acute inflammatory responses and appeared to reduce the incidence of heart failure [148,149]. In patients with heart failure with reduced or preserved ejection fraction, anakinra given for 14 days significantly improved exercise capacity, measured as peak oxygen consumption [150,151]. IL-1β blockade with canakinumab is also under investigation in a large clinical trial of 10,000 patients with prior AMI [152]. The effects of these pharmacologic agents on ischemia-reperfusion injury following heart transplant or in heart transplant rejection are however unknown. 

## 9. Conclusions 

Heart transplantation success is influenced by the intrinsic damage linked to the ischemic and inflammatory injuries during all phases of organ procurement and transplantation. Unfortunately, myocardial injury starts in the donor and continues in the recipient. Understanding the mechanisms of the innate immune responses is vital to reduce graft injury and corroborate the strategies to reduce activation of the adaptive immunity. Progress toward this knowledge will support the development of targeted therapies to be used to preserve or minimize the myocardial injury during heart transplantation, and also to potentially increase the pool of transplantable donor hearts by considering DCD hearts. A better understanding of the innate immune response of the transplanted heart may lead to novel therapeutic strategies to protect the graft from ischemic and inflammatory injury. 

## Figures and Tables

**Table 1:** Abbreviations.

Abbreviations	Full Names
AAT	Alpha-1Antitrypsin
ACR	Acute cellular rejection
AdorA2B	Adenosine Receptor A2B
AMI	Acute Myocardial Infarction
AMR	Antibody-mediated rejection
APC	Antigen Presenting Cells
ASC	Apoptosis Speck-Like Protein containing a Caspase recruiting domain (CARD)
ATP	Adenosine Triphosphate
β-AR	β-Adrenergic Receptor
CARD	Caspase recruiting domain
CAV	Coronary Artery Vasculopathy
DAMPS	Damage Associated Molecular Patterns
DBD	Donation after Brain Death
DCD	Donation after Cardiac Death
HF	Heart Failure
HMGB-1	High-mobility group protein B1
HMP	Hypothermic Machine Perfusion
HTx	Heart Transplantation
IL-1	Interleukin-1
IL-18BP	Interleukin-18 Binding Protein
IL-1Ra	IL-1 receptor antagonist
IL-1RAp	IL-1R Accessory protein
IL-1RI	Interleukin-1 receptor type 1
IRFs	Interferon Regulated Transcription Factors
ISHLT	International Society of Heart and Lung Transplantation
LRRs	Leucine-rich repeats
LVADs	Left Ventricular Assisting Devices
MAPK	Mitogen Activated Protein Kinases
MHC	Major Histocompatibility Complex
MyD88	Myeloid Differentiation Factor 88
NADPH	Nicotinamide Adenine Dinucleotide Phosphate
NF-kB	Nuclear Factor-κB
NLRP3	NOD Like Receptors (NLR) containing a Pyrin Domain
PGD	Primary Graft Dysfunction
PRRs	Pattern Recognition Receptors
P2X7R	Purinergic 2X Receptor 7
PYD	PYRIN Domain
ROS	Reactive Oxygen Species
SCS	Static Cold Storage
TIR	Toll/Interleukin-1 Receptor
TIRAP	Toll/Interleukin-1 Receptor (TIR) domain containing an adaptor protein
TLR	Toll-Like Receptor
TNF-α	Tumor Necrosis Factor-alpha
TRAP	Toll/Interleukin-1 Receptor (TIR) Adaptor Protein
TRIF	TIR-domain-containing adapter-inducing interferon-β

## References

[1] Mancini D., Lietz K. (2010). Selection of cardiac transplantation candidates in 2010. Circulation.

[2] American Heart Association. http://www.heart.org/HEARTORG/Conditions/HeartFailure/Advanced-Heart-Failure_UCM_441925_Article.jsp.

[3] Benden C., Goldfarb S.B., Edwards L.B., Kucheryavaya A.Y., Christie J.D., Dipchand A.I., Dobbels F., Levvey B.J., Lund L.H., Meiser B. (2014). The registry of the International Society for Heart and Lung Transplantation: Seventeenth official pediatric lung and heart-lung transplantation report—2014; Focus theme: Retransplantation. J. Heart Lung Transpl..

[4] Mozaffarian D., Benjamin E.J., Go A.S., Arnett D.K., Blaha M.J., Cushman M., de Ferranti S., Després J.P., Fullerton H.J., Howard V.J. (2015). Heart disease and stroke statistics—2015 Update: A report from the American Heart Association. Circulation.

[5] Roger V.L. (2013). Epidemiology of heart failure. Circ. Res..

[6] Braunwald E. (2015). The war against heart failure: The Lancet lecture. Lancet.

[7] Smolina K., Wright F.L., Rayner M., Goldacre M.J. (2012). Determinants of the decline in mortality from acute myocardial infarction in England between 2002 and 2010: Linked national database study. BMJ.

[8] Colvin-Adams M., Smithy J.M., Heubner B.M., Skeans M.A., Edwards L.B., Waller C., Schnitzler M.A., Snyder J.J., Israni A.K., Kasiske B.L. (2014). OPTN/SRTR 2012 Annual Data Report: Heart. Am. J. Transpl..

[9] Neyrinck A., van Raemdonck D., Monbaliu D. (2013). Donation after circulatory death: Current status. Curr. Opin. Anaesthesiol..

[10] Muralidharan S., Mandrekar P. (2013). Cellular stress response and innate immune signaling: Integrating pathways in host defense and inflammation. J. Leukoc. Biol..

[11] Saidi R.F., Bradley J., Greer D., Luskin R., O’Connor K., Delmonico F., Kennealey P., Pathan F., Schuetz C., Elias N. (2010). Changing pattern of organ donation at a single center: Are potential brain dead donors being lost to donation after cardiac death?. Am. J. Transpl..

[12] Laks H., Scholl F.G., Drinkwater D.C., Blitz A., Hamilton M., Moriguchi J., Fonarow G., Kobashigawa J. (1997). The alternate recipient list for heart transplantation: Does it work?. J. Heart Lung Transpl..

[13] Chen J.M., Russo M.J., Hammond K.M., Mancini D.M., Kherani A.R., Fal J.M., Mazzeo P.A., Pinney S.P., Edwards N.M., Naka Y. (2005). Alternate waiting list strategies for heart transplantation maximize donor organ utilization. Ann. Thorac. Surg..

[14] Laks H., Marelli D., Fonarow G.C., Hamilton M.A., Ardehali A., Moriguchi J.D., Bresson J., Gjertson D., Kobashigawa J.A., UCLA Heart Tranplant Group (2003). Use of two recipient lists for adults requiring heart transplantation. J. Thorac. Cardiovasc. Surg..

[15] Poston R.S., Griffith B.P. (2004). Heart transplantation. J. Intensive Care Med..

[16] López-Navidad A., Caballero F. (2003). Extended criteria for organ acceptance. Strategies for achieving organ safety and for increasing organ pool. Clin. Transpl..

[17] Wittwer T., Wahlers T. (2008). Marginal donor grafts in heart transplantation: Lessons learned from 25 years of experience. Transpl. Int..

[18] Orioles A., Morrison W.E., Rossano J.W., Shore P.M., Hasz R.D., Martiner A.C., Berg R.A., Nadkarni V.M. (2013). An under-recognized benefit of cardiopulmonary resuscitation: Organ transplantation. Crit. Care Med..

[19] Quader M.A., Wolfe L.G., Kasirajan V. (2013). Heart transplantation outcomes from cardiac arrest-resuscitated donors. J. Heart Lung Transpl..

[20] Quader M., Wolfe L., Katlaps G., Kasirajan V. (2014). Donor heart utilization following cardiopulmonary arrest and resuscitation: Influence of donor characteristics and wait times in transplant regions. J. Transpl..

[21] Blackstock M.J., Ray D.C. (2014). Organ donation after circulatory death: An update. Eur. J. Emerg. Med..

[22] Morrissey P.E., Monaco A.P. (2014). Donation after circulatory death: Current practices, ongoing challenges, and potential improvements. Transplantation.

[23] Pomfret E.A., Sung R.S., Allan J., Kinkhabwala M., Melancon J.K., Roberts J.P. (2008). Solving the organ shortage crisis: The 7th annual American Society of Transplant Surgeons’ State-of-the-Art Winter Symposium. Am. J. Transpl..

[24] Osaki S., Locher M.R., Lushaj E.B., Akhter S.A., Kohmoto T. (2014). Functional evaluation of human donation after cardiac death donor hearts using a continuous isolated myocardial perfusion technique: Potential for expansion of the cardiac donor population. J. Thorac. Cardiovasc. Surg..

[25] Stadelmann M., Dornbierer M., Clément D., Gahl B., Dick F., Carrel T.P., Tevaearai H.T., Longnus S. (2013). Mild hypothermia during global cardiac ischemia opens a window of opportunity to develop heart donation after cardiac death. Transpl. Int..

[26] Gries C.J., White D.B., Truog R.D., Dubois J., Cosio C.C., Dhanani S., Chan K.M., Corris P., Dark J., Fulda G. (2013). An official American Thoracic Society/International Society for Heart and Lung Transplantation/Society of Critical Care Medicine/Association of Organ and Procurement Organizations/United Network of Organ Sharing Statement: Ethical and policy considerations in organ donation after circulatory determination of death. Am. J. Respir. Crit. Care Med..

[27] Dalle Ave A.L., Shaw D.M., Pascual M., Benaroyo L. (2014). Heart donation after circulatory determination of death: Ethically acceptable?. Nat. Rev. Cardiol..

[28] Osaki S., Anderson J.E., Johnson M.R., Edwards N.M., Kohmoto T. (2010). The potential of cardiac allografts from donors after cardiac death at the University of Wisconsin Organ Procurement Organization. Eur. J. Cardiothorac. Surg..

[29] Noterdaeme T., Detry O., Hans M.F., Nellessen E., Ledoux D., Joris J., Meurisse M., Defraigne J.O. (2013). What is the potential increase in the heart graft pool by cardiac donation after circulatory death?. Transpl. Int..

[30] Birati E.Y., Rame J.E. (2014). Left ventricular assist device management and complications. Crit. Care Clin..

[31] Mancini D., Colombo P.C. (2015). Left ventricular assist devices: A rapidly evolving alternative to transplant. J. Am. Coll. Cardiol..

[32] Stevenson L.W., Rose E.A. (2003). Left ventricular assist devices: Bridges to transplantation, recovery, and destination for whom?. Circulation.

[33] Jaski B.E., Kim J.C., Naftel D.C., Jarcho J., Costanzo M.R., Eisen H.J., Kirklin J.K., Bourge R.C., Cardiac Transplant Research Database Research Group (2001). Cardiac transplant outcome of patients supported on left ventricular assist device *vs.* intravenous inotropic therapy. J. Heart Lung Transpl..

[34] Dhital K.K., Iyer A., Connellan M., Chew H.C., Gao L., Doyle A., Hicks M., Kumarasinghe G., Soto C., Dinale A. (2015). Adult heart transplantation with distant procurement and *ex vivo* preservation of donor hearts after circulatory death: A case series. Lancet.

[35] McKeown D.W., Bonser R.S., Kellum J.A. (2012). Management of the heartbeating brain-dead organ donor. Br. J. Anaesth..

[36] Mackersie R., Bronsther O., Shackford S. (1991). Organ procurement in patients with fatal head injuries. The fate of the potential donor. Ann. Surg..

[37] Bugge J. (2009). Brain death and its implications for management of the potential organ donor. Acta Anaesthesiol. Scand..

[38] Smith M. (2004). Physiologic changes during brain stem death—Lessons for management of the organ donor. J. Heart Lung Transpl..

[39] Marasco S.F., Kras A., Schulberg E., Vale M., Lee G.A. (2012). Impact of warm ischemia time on survival after heart transplantation. Transpl. Proc..

[40] Mitropoulos F.A., Odim J., Marelli D., Karandikar K., Gjertson D., Ardehali A., Kobashigawa J., Laks H. (2005). Outcome of hearts with cold ischemic time greater than 300 min. A case-matched study. Eur. J. Cardiothorac. Surg..

[41] Guibert E.E., Petrenko A.Y., Balaban C.L., Somov A.Y., Rodriguez J.V., Fuller B.J. (2011). Organ preservation: Current concepts and new strategies for the next decade. Transfus. Med. Hemother..

[42] McAnulty J.F. (2010). Hypothermic organ preservation by static storage methods: Current status and a view to the future. Cryobiology.

[43] Fuster V., Badimon L., Badimon J.J., Chesebro J.H. (1992). The pathogenesis of coronary artery disease and the acute coronary syndromes. N. Engl. J. Med..

[44] Jennings R.B., Reimer K.A., Steenbergen C. (1986). Myocardial ischemia revisited. The osmolar load, membrane damage, and reperfusion. J. Mol. Cell. Cardiol..

[45] Fuller B., Guibert E., Rodriguez J., Lubens E., Cerda J., Clark M. (2010). Lessons from natural cold-induced dormancy to organ preservation in medicine and biotechnology: From the ‘backwoods to the bedside’. Dormancy and Resistance to Harsh Environments.

[46] Rauen U., de Groot H. (2004). New insights into the cellular and molecular mechanisms of cold storage injury. J. Investig. Med..

[47] Hosgood S.A., Bagul A., Nicholson M.L. (2010). Minimising cold ischaemic injury in an experimental model of kidney transplantation. Eur. J. Clin. Investig..

[48] Del Rizzo D.F., Menkis A.H., Pflugfelder P.W., Novick R.J., McKenzie F.N., Boyd W.D., Kostuk W.J. (1999). The role of donor age and ischemic time on survival following orthotopic heart transplantation. J. Heart Lung Transpl..

[49] Southard J., Belzer F.O. (1995). Organ preservation. Annu. Rev. Med..

[50] Desrois M., Piccardo A., Zogheib E., Dalmasso C., Lan C., Fourré D., Cozzone P.J., Caus T., Bernard M. (2014). Heart donation after cardiac death: Preliminary study on an isolated, perfused swine heart after 20 min of normothermic ischemia. Transpl. Proc..

[51] Van Caenegem O., Beauloye C., Bertrand L., Horman S., Lepropre S., Sparavier G., Vercruysse J., Bethuyne N., Poncelet A.J., Gianello P. (2015). Hypothermic continuous machine perfusion enables preservation of energy charge and functional recovery of heart grafts in an *ex vivo* model of donation following circulatory death. Eur. J. Cardiothorac. Surg..

[52] Niemann C.U., Feiner J., Swain S., Bunting S., Friedman M., Crutchfield M., Broglio K., Hirose R., Roberts J.P., Malinoski D. (2015). Therapeutic hypothermia in deceased organ donors and kidney-graft function. N. Engl. J. Med..

[53] Hicks M., Hing A., Gao L., Ryan J., Macdonald P.S. (2006). Organ preservation. Methods Mol. Biol..

[54] Barry W. (1987). Mechanisms of myocardial cell injury during ischemia and reperfusion. J. Card. Surg..

[55] Seccombe J.F., Schaff H.V. (1995). Coronary artery endothelial function after myocardial ischemia and reperfusion. Ann. Thorac. Surg..

[56] Hausenloy D.J., Yellon D.M. (2013). Myocardial ischemia-reperfusion injury: A neglected therapeutic target. J. Clin. Investig..

[57] Verma S., Fedak P.W., Weisel R.D., Butany J., Rao V., Maitland A., Li R.K., Dhillon B., Yau T.M. (2002). Fundamentals of reperfusion injury for the clinical cardiologist. Circulation.

[58] Kalogeris T., Bao Y., Korthuis R.J. (2014). Mitochondrial reactive oxygen species: A double edged sword in ischemia/reperfusion *vs.* preconditioning. Redox Biol..

[59] Gottlieb R.A., Burleson K.O., Kloner R.A., Babior B.M., Engler R.L. (1994). Reperfusion injury induces apoptosis in rabbit cardiomyocytes. J. Clin. Investig..

[60] Marchant D.J., Boyd J.H., Lin D.C., Granville D.J., Garmaroudi F.S., McManus B.M. (2012). Inflammation in myocardial diseases. Circ. Res..

[61] Ali A.A., White P., Xiang B., Lin H.Y., Tsui S.S., Ashley E., Lee T.W., Klein J.R., Kumar K., Arora R.C. (2011). Hearts from DCD donors display acceptable biventricular function after heart transplantation in pigs. Am. J. Transpl..

[62] Neumar R.W., Nolan J.P., Adrie C., Aibiki M., Berg R.A., Böttiger B.W., Callaway C., Clark R.S., Geocadin R.G., Jauch E.C. (2008). Post-cardiac arrest syndrome: Epidemiology, pathophysiology, treatment, and prognostication. A consensus statement from the International Liaison Committee on Resuscitation (American Heart Association, Australian and New Zealand Council on Resuscitation, European Resuscitation Council, Heart and Stroke Foundation of Canada, InterAmerican Heart Foundation, Resuscitation Council of Asia, and the Resuscitation Council of Southern Africa); the American Heart Association Emergency Cardiovascular Care Committee; the Council on Cardiovascular Surgery and Anesthesia; the Council on Cardiopulmonary, Perioperative, and Critical Care; the Council on Clinical Cardiology; and the Stroke Council. Circulation.

[63] Iyer A., Kumarasinghe G., Hicks M., Watson A., Gao L., Doyle A., Keogh A., Kotlyar E., Hayward C., Dhital K. (2011). Primary graft failure after heart transplantation. J. Transpl..

[64] Zipes D.P., Wellens H.J. (1998). Sudden cardiac death. Circulation.

[65] Singh D., Taylor D.O. (2015). Advances in the understanding and management of heart transplantation. F1000Prime Rep..

[66] Shen H., Kreisel D., Goldstein D.R. (2013). Processes of Sterile Inflammation. J. Immunol..

[67] LaRosa D.F., Rahman A.H., Turka L.A. (2007). The innate immune system in allograft rejection and tolerance. J. Immunol..

[68] Arslan F., de Kleijn D.P., Pasterkamp G. (2011). Innate immune signaling in cardiac ischemia. Nat. Rev. Cardiol..

[69] Toldo S., Mezzaroma E., Mauro A.G., Salloum F., van Tassell B.W., Abbate A. (2015). The inflammasome in myocardial injury and cardiac remodeling. Antioxid. Redox Signal..

[70] Zedler S., Faist E. (2006). The impact of endogenous triggers on trauma-associated inflammation. Curr. Opin. Crit. Care.

[71] Mariathasan S., Monack D.M. (2007). Inflammasome adaptors and sensors: Intracellular regulators of infection and inflammation. Nat. Rev. Immunol..

[72] Netea M.G., Nold-Petry C.A., Nold M.F., Joosten L.A., Opitz B., van der Meer J.H., van de Veerdonk F.L., Ferwerda G., Heinhuis B., Devesa I. (2009). Differential requirement for the activation of the inflammasome for processing and release of IL-1beta in monocytes and macrophages. Blood.

[73] Van Tassell B.W., Toldo S., Mezzaroma E., Abbate A. (2013). Targeting interleukin-1 in heart disease. Circulation.

[74] Kawaguchi M., Takahashi M., Hata T., Kashima Y., Usui F., Morimoto H., Izawa A., Takahashi Y., Masumoto J., Koyama J. (2011). Inflammasome Activation of Cardiac Fibroblasts Is Essential for Myocardial Ischemia/Reperfusion Injury. Circulation.

[75] Mezzaroma E., Toldo S., Farkas D., Seropian I.M., van Tassell B.W., Salloum F.N., Kannan H.R., Menna A.C., Voelkel N.F., Abbate A. (2011). The inflammasome promotes adverse cardiac remodeling following acute myocardial infarction in the mouse. Proc. Natl. Acad. Sci. USA.

[76] Sandanger Ø., Ranheim T., Vinge L.E., Bliksøen M., Alfsnes K., Finsen A.V., Dahl C.P., Askevold E.T., Florholmen G., Christensen G. (2013). The NLRP3 inflammasome is up-regulated in cardiac fibroblasts and mediates myocardial ischaemia-reperfusion injury. Cardiovasc. Res..

[77] Bracey N.A., Beck P.L., Muruve D.A., Hirota S.A., Guo J., Jabagi H., Wright J.R., Macdonald J.A., Lees-Miller J.P., Roach D. (2013). The Nlrp3 inflammasome promotes myocardial dysfunction in structural cardiomyopathy through interleukin-1β. Exp. Physiol..

[78] Toldo S., Kannan H., Bussani R., Anzini M., Sonnino C., Sinagra G., Merlo M., Mezzaroma E., De-Giorgio F., Silvestri F. (2014). Formation of the inflammasome in acute myocarditis. Int. J. Cardiol..

[79] Toldo S., Mezzaroma E., Abbate A. (2012). Interleukin-1 Blockade in Acute Myocardial Infarction and Heart Failure: Ready for Clinical Translation?. Transl. Med..

[80] Lund L.H., Edwards L.B., Kucheryavaya A.Y., Dipchand A.I., Benden C., Christie J.D., Dobbels F., Kirk R., Rahmel A.O., Yusen R.D. (2013). The Registry of the International Society for Heart and Lung Transplantation: Thirtieth official adult heart transplant report—2013; Focus theme: Age. J. Heart Lung Transpl..

[81] Costanzo M.R., Dipchand A., Starling R., Anderson A., Chan M., Desai S., Fedson S., Fisher P., Gonzales-Stawinski G., Martinelli L. (2010). The International Society of Heart and Lung Transplantation Guidelines for the care of heart transplant recipients. J. Heart Lung Transpl..

[82] Chen E.P., Bittner H.B., Kendall S.W., van Trigt P. (1996). Hormonal and hemodynamic changes in a validated animal model of brain death. Crit. Care Med..

[83] Atkinson C., Varela J.C., Tomlinson S. (2009). Complement-dependent inflammation and injury in a murine model of brain dead donor hearts. Circ. Res..

[84] Atkinson C., Floerchinger B., Qiao F., Casey S., Williamson T., Moseley E., Stoica S., Goddard M., Ge X., Tullius S.G. (2013). Donor brain death exacerbates complement-dependent ischemia/reperfusion injury in transplanted hearts. Circulation.

[85] Danobeitia J.S., Djamali A., Fernandez L.A. (2014). The role of complement in the pathogenesis of renal ischemia-reperfusion injury and fibrosis. Fibrogenes. Tissue Repair.

[86] Damman J., Hoeger S., Boneschansker L., Theruvath A., Waldherr R., Leuvenink H.G., Ploeg R.J., Yard B.A., Seelen M.A. (2011). Targeting complement activation in brain-dead donors improves renal function after transplantation. Transpl. Immunol..

[87] Floerchinger B., Yuan X., Jurisch A., Timsit M.O., Ge X., Lee Y.L., Schmid C., Tullius S.G. (2012). Inflammatory immune responses in a reproducible mouse brain death model. Transpl. Immunol..

[88] Cannon J.G., Evans W.J., Hughes V.A., Meredith C.N., Dinarello C.A. (1986). Physiological mechanisms contributing to increased interleukin-1 secretion. J. Appl. Physiol..

[89] Van der Poll T., Lowry S.F. (1997). Epinephrine inhibits endotoxin-induced IL-1 beta production: Roles of tumor necrosis factor-alpha and IL-10. Am. J. Physiol..

[90] Takada M., Nadeau K.C., Shaw G.D., Marquette K.A., Tilney N.L. (1997). The cytokine-adhesion molecule cascade in ischemia/reperfusion injury of the rat kidney. Inhibition by a soluble P-selectin ligand. J. Clin. Investig..

[91] Zhu H., Li J., Wang S., Liu K., Wang L., Huang L. (2013). Hmgb1-TLR4-IL-23-IL-17A axis promote ischemia-reperfusion injury in a cardiac transplantation model. Transplantation.

[92] Dare A.J., Logan A., Prime T.A., Rogatti S., Goddard M., Bolton E.M., Bradley J.A., Pettigrew G.J., Murphy M.P., Saeb-Parsy K. (2015). The mitochondria-targeted anti-oxidant MitoQ decreases ischemia-reperfusion injury in a murine syngeneic heart transplant model. J. Heart Lung Transpl..

[93] Wenceslau C.F., McCarthy C.G., Szasz T., Spitler K., Goulopoulou S., Webb R.C., Working Group on DAMPs in Cardiovascular Disease (2014). Mitochondrial damage-associated molecular patterns and vascular function. Eur. Heart J..

[94] Martinon F. (2010). Signaling by ROS drives inflammasome activation. Eur. J. Immunol..

[95] Oka T., Hikoso S., Yamaguchi O., Taneike M., Takeda T., Tamai T., Oyabu J., Murakawa T., Nakayama H., Nishida K. (2012). Mitochondrial DNA that escapes from autophagy causes inflammation and heart failure. Nature.

[96] Tozzi M., Franchin M., Soldini G., Ietto G., Chiappa C., Maritan E., Villa F., Carcano G., Dionigi R. (2013). Impact of static cold storage VS hypothermic machine preservation on ischemic kidney graft: Inflammatory cytokines and adhesion molecules as markers of ischemia/reperfusion tissue damage. Our preliminary results. Int. J. Surg..

[97] Christia P., Frangogiannis N.G. (2013). Targeting inflammatory pathways in myocardial infarction. Eur. J. Clin. Investig..

[98] Frangogiannis N.G. (2014). The inflammatory response in myocardial injury, repair, and remodelling. Nat. Rev. Cardiol..

[99] Kobashigawa J., Zuckermann A., Macdonald P., Leprince P., Esmailian F., Luu M., Mancini D., Patel J., Razi R., Reichenspurner H. (2014). Report from a consensus conference on primary graft dysfunction after cardiac transplantation. J. Heart Lung Transpl..

[100] Chew H.C., Kumarasinghe G., Iyer A., Hicks M., Gao L., Doyle A., Jabbour A., Dhital K., Granger E., Jansz P. (2014). Primary Graft Dysfunction After Heart Transplantation. Curr. Transpl. Rep..

[101] O’Brien L.C., Mezzaroma E., van Tassell B.W., Marchetti C., Carbone S., Abbate A., Toldo S. (2014). Interleukin-18 as a therapeutic target in acute myocardial infarction and heart failure. Mol. Med..

[102] Crudele V., Picascia A., Infante T., Grimaldi V., Maiello C., Napoli C. (2011). Repeated immune and non immune insults to the graft after heart transplantation. Immunol. Lett..

[103] Afzali B., Lechler R.I., Hernandez-Fuentes M.P. (2007). Allorecognition and the allore-sponse: Clinical implications. Tissue Antigens.

[104] Gallon L.G., Leventhal J.R., Kaufman D.B. (2002). Pretransplantevaluation of renal transplant candidates. Semin. Nephrol..

[105] Stewart S., Winters G.L., Fishbein M.C., Tazelaar H.D., Kobashigawa J., Abrams J., Andersen C.B., Angelini A., Berry G.J., Burke M.M. (2005). Revision of the 1990 working formulation for the standardization of nomenclature in the diagnosis of heart rejection. J. Heart Lung Transpl..

[106] Gass A.L., Emaminia A., Lanier G., Aggarwal C., Brown K.A., Raffa M., Kai M., Spielvogel D., Malekan R., Tang G. (2015). Cardiac Transplantation in the New Era. Cardiol. Rev..

[107] Chih S., Chruscinski A., Ross H.J., Tinckam K., Butany J., Rao V. (2012). Antibody-mediated rejection: An evolving entity in heart transplantation. J. Transpl..

[108] You S. (2015). Differential sensitivity of regulatory and effector T cells to cell death: A prerequisite for transplant tolerance. Front. Immunol..

[109] Rao D.A., Eid R.E., Qin L., Yi T., Kirkiles-Smith N.C., Tellides G., Pober J.S. (2008). Interleukin (IL)-1 promotes allogeneic T cell intimal infiltration and IL-17 production in a model of human artery rejection. J. Exp. Med..

[110] Kumar H., Kawai T., Akira S. (2009). Toll-like receptors and innate immunity. Biochem. Biophys. Res. Commun..

[111] Kaczorowski D.J., Nakao A., McCurry K.R., Billiar T.R. (2009). Toll-like receptors and myocardial ischemia/reperfusion, inflammation, and injury. Curr. Cardiol..

[112] Vilahur G., Badimon L. (2014). Ischemia/reperfusion activates myocardial innate immune response: The key role of the Toll-like receptor. Front. Physiol..

[113] Weber D.J., Gracon A.S., Ripsch M.S., Fisher A.J., Cheon B.M., Pandya P.H., Vittal R., Capitano M.L., Kim Y., Allette Y.M. (2014). The HMGB1-RAGE axis mediates traumatic brain injury-induced pulmonary dysfunction in lung transplantation. Sci. Transl. Med..

[114] Stribos E.G., van Werkhoven M.B., Poppelaars F., van Goor H., Olinga P., van Son W.J., Damman J., Seelen M.A. (2015). Renal expression of Toll-like receptor 2 and 4: Dynamics in human allograft injury and comparison to rodents. Mol. Immunol..

[115] Krüger B., Krick S., Dhillon N., Lerner S.M., Ames S., Bromberg J.S., Lin M., Walsh L., Vella J., Fischereder M. (2009). Donor Toll-like receptor 4 contributes to ischemia and reperfusion injury following human kidney transplantation. Proc. Natl. Acad. Sci. USA.

[116] Hoffmann U., Bergler T., Rihm M., Pace C., Krüger B., Jung B., Reinhold S.W., Farkas S., Rümmele P., Krämer B.K. (2011). Impact of Toll-like receptor 2 expression in renal allograft rejection. Nephrol. Dial. Transpl..

[117] Zhang X., Beduhn M., Zheng X., Lian D., Chen D., Li R., Siu L.K., Marleau A., French P.W., Ichim T.E. (2012). Induction of alloimmune tolerance in heart transplantation through gene silencing of TLR adaptors. Am. J. Transpl..

[118] Wang S., Schmaderer C., Kiss E., Schmidt C., Bonrouhi M., Porubsky S., Gretz N., Schaefer L., Kirschning C.J., Popovic Z.V. (2010). Recipient Toll-like receptors contribute to chronic graft dysfunction by both MyD88- and TRIF-dependent signaling. Dis. Model. Mech..

[119] Toldo S., Mezzaroma E., McGeough M.D., Peña C.A., Marchetti C., Sonnino C., van Tassell B.W., Salloum F.N., Voelkel N.F., Hoffman H.M. (2015). Independent roles of the priming and the triggering of the NLRP3 inflammasome in the heart. Cardiovasc. Res..

[120] Takahashi M. (2011). NLRP3 Inflammasome as a Novel Player in Myocardial Infarction. Int. Heart J..

[121] Toldo S., Zhong H., Mezzaroma E., van Tassell B.W., Kannan H., Zeng D., Belardinelli L., Voelkel N.F., Abbate A. (2012). GS-6201, a selective blocker of the A2B adenosine receptor, attenuates cardiac remodeling after acute myocardial infarction in the mouse. J. Pharmacol. Exp. Ther..

[122] Gu H., Xie M., Xu L., Zheng X., Yang Y., Lv X. (2015). The protective role of interleukin-18 binding protein in a murine model of cardiac ischemia/reperfusion injury. Transpl. Int..

[123] Jankovic D., Ganesan J., Bscheider M., Stickel N., Weber F.C., Guarda G., Follo M., Pfeifer D., Tardivel A., Ludigs K. (2013). The Nlrp3 inflammasome regulates acute graft-versus-host disease. J. Exp. Med..

[124] Seto T., Kamijo S., Wada Y., Yamaura K., Takahashi K., Komatsu K., Otsu Y., Terasaki T., Fukui D., Amano J. (2010). Upregulation of the apoptosis-related inflammasome in cardiac allograft rejection. J. Heart Lung Transpl..

[125] Shah K.B., Mauro A.G., Flattery M., Toldo S., Abbate A. (2015). Formation of the inflammasome during cardiac allograft rejection. Int. J. Cardiol..

[126] Marasco S.F., Sheeran F.L., Chaudhuri K., Vale M., Bailey M., Pepe S. (2014). Molecular markers of programmed cell death in donor hearts before transplantation. J. Heart Lung Transpl..

[127] Baldwin A.G., Brough D., Freeman S. (2016). Inhibiting the Inflammasome: A Chemical Perspective. J. Med. Chem..

[128] Marchetti C., Chojnacki J., Toldo S., Mezzaroma E., Tranchida N., Rose S.W., Federici M., van Tassell B.W., Zhang S., Abbate A. (2014). A Novel Pharmacologic Inhibitor of the NLRP3 Inflammasome Limits Myocardial Injury after Ischemia-Reperfusion in the Mouse. J. Cardiovasc. Pharmacol..

[129] Marchetti C., Toldo S., Chojnacki J., Mezzaroma E., Liu K., Salloum F.N., Nordio A., Carbone S., Mauro A.G., Das A. (2015). Pharmacologic Inhibition of the NLRP3 Inflammasome Preserves Cardiac Function After Ischemic and Nonischemic Injury in the Mouse. J. Cardiovasc. Pharmacol..

[130] Toldo S., Marchetti C., Mauro A.G., Chojnacki J., Mezzaroma E., Carbone S., Zhang S., van Tassell B., Salloum F.N., Abbate A. (2016). Inhibition of the NLRP3 inflammasome limits the inflammatory injury following myocardial ischemia-reperfusion in the mouse. Int. J. Cardiol..

[131] Coll R.C., Robertson A.A., Chae J.J., Higgins S.C., Muñoz-Planillo R., Inserra M.C., Vetter I., Dungan L.S., Monks B.G., Stutz A. (2015). A small-molecule inhibitor of the NLRP3 inflammasome for the treatment of inflammatory diseases. Nat. Med..

[132] Bartlett R., Stokes L., Sluyter R. (2014). The P2X7 receptor channel: Recent developments and the use of P2X7 antagonists in models of disease. Pharmacol. Rev..

[133] Polosa R., Blackburn M.R. (2009). Adenosine receptors as targets for therapeutic intervention in asthma and chronic obstructive pulmonary disease. Trends Pharmacol. Sci..

[134] Toldo S., Seropian I.M., Mezzaroma E., van Tassell B.W., Salloum F.N., Lewis E.C., Voelkel N., Dinarello C.A., Abbate A. (2011). Alpha-1 antitrypsin inhibits caspase-1 and protects from acute myocardial ischemia-reperfusion injury. J. Mol. Cell. Cardiol..

[135] Toldo S., Mauro A.G., Marchetti C., Gelber C., Mezzaroma E., Wolpe S., Yachin G., Salloum F.N., van Tassell B., Abbate A. (2015). Anti-inflammatory peptide SP16 reduces infarct size after myocardial ischemia and reperfusion in the mouse. Eur. Heart J..

[136] Toldo S., Das A., Mezzaroma E., Chau V.Q., Marchetti C., Durrant D., Samidurai A., van Tassell B.W., Yin C., Ockaili R.A. (2014). Induction of microRNA-21 with exogenous hydrogen sulfide attenuates myocardial ischemic and inflammatory injury in mice. Circ. Cardiovasc. Genet..

[137] Liu Y., Lian K., Zhang L., Wang R., Yi F., Gao C., Xin C., Zhu D., Li Y., Yan W. (2014). TXNIP mediates NLRP3 inflammasome activation in cardiac microvascular endothelial cells as a novel mechanism in myocardial ischemia/reperfusion injury. Basic Res. Cardiol..

[138] Hoffman H.M., Throne M.L., Amar N.J., Sebai M., Kivitz A.J., Kavanaugh A., Weinstein S.P., Belomestnov P., Yancopoulos G.D., Stahl N. (2008). Efficacy and safety of rilonacept (interleukin-1 Trap) in patients with cryopyrin-associated periodic syndromes: Results from two sequential placebo-controlled studies. Arthritis Rheumatol..

[139] Lachmann H.J., Kone-Paut I., Kuemmerle-Deschner J.B., Leslie K.S., Hachulla E., Quartier P., Gitton X., Widmer A., Patel N., Hawkins P.N. (2009). Use of canakinumab in the cryopyrin-associated periodic syndrome. N. Engl. J. Med..

[140] Neven B., Marvillet I., Terrada C., Ferster A., Boddaert N., Couloignier V., Pinto G., Pagnier A., Bodemer C., Bodaghi B. (2010). Long-term efficacy of the interleukin-1 receptor antagonist anakinra in ten patients with neonatal-onset multisystem inflammatory disease/chronic infantile neurologic, cutaneous, articular syndrome. Arthritis Rheumatol..

[141] Toldo S., Mezzaroma E., van Tassell B.W., Farkas D., Marchetti C., Voelkel N.F., Abbate A. (2013). Interleukin-1β blockade improves cardiac remodelling after myocardial infarction without interrupting the inflammasome in the mouse. Exp. Physiol..

[142] Toldo S., Schatz A.M., Mezzaroma E., Chawla R., Stallard T.W., Stallard W.C., Jahangiri A., van Tassell B.W., Abbate A. (2012). Recombinant human interleukin-1 receptor antagonist provides cardioprotection during myocardial ischemia reperfusion in the mouse. Cardiovasc. Drugs Ther..

[143] Toldo S., Mezzaroma E., Bressi E., Marchetti C., Carbone S., Sonnino C., van Tassell B.W., Abbate A. (2014). Interleukin-1β blockade improves left ventricular systolic/diastolic function and restores contractility reserve in severe ischemic cardiomyopathy in the mouse. J. Cardiovasc. Pharmacol..

[144] Abbate A., van Tassell B.W., Seropian I.M., Toldo S., Robati R., Varma A., Salloum F.N., Smithson L., Dinarello C.A. (2010). Interleukin-1beta modulation using a genetically engineered antibody prevents adverse cardiac remodelling following acute myocardial infarction in the mouse. Eur. J. Heart Fail..

[145] Van Tassell B.W., Varma A., Salloum F.N., Das A., Seropian I.M., Toldo S., Smithson L., Hoke N.N., Chau V.Q., Robati R. (2010). Interleukin-1 trap attenuates cardiac remodeling after experimental acute myocardial infarction in mice. J. Cardiovasc. Pharmacol..

[146] Salloum F.N., Chau V., Varma A., Hoke N.N., Toldo S., Biondi-Zoccai G.G., Crea F., Vetrovec G.W., Abbate A. (2009). Anakinra in experimental acute myocardial infarction—Does dosage or duration of treatment matter?. Cardiovasc. Drugs Ther..

[147] Abbate A., Salloum F.N., Vecile E., Das A., Hoke N.N., Straino S., Biondi-Zoccai G.G., Houser J.E., Qureshi I.Z., Ownby E.D. (2008). Anakinra, a recombinant human interleukin-1 receptor antagonist, inhibits apoptosis in experimental acute myocardial infarction. Circulation.

[148] Abbate A., van Tassell B.W., Biondi-Zoccai G., Kontos M.C., Grizzard J.D., Spillman D.W., Oddi C., Roberts C.S., Melchior R.D., Mueller G.H. (2013). Effects of interleukin-1 blockade with anakinra on adverse cardiac remodeling and heart failure after acute myocardial infarction [from the Virginia Commonwealth University-Anakinra Remodeling Trial (2) (VCU-ART2) pilot study]. Am. J. Cardiol..

[149] Abbate A., Kontos M.C., Grizzard J.D., Biondi-Zoccai G.G., van Tassell B.W., Robati R., Roach L.M., Arena R.A., Roberts C.S., Varma A. (2010). Interleukin-1 blockade with anakinra to prevent adverse cardiac remodeling after acute myocardial infarction (Virginia Commonwealth University Anakinra Remodeling Trial [VCU-ART] Pilot study). Am. J. Cardiol..

[150] Van Tassell B.W., Arena R.A., Toldo S., Mezzaroma E., Azam T., Seropian I.M., Shah K., Canada J., Voelkel N.F., Dinarello C.A. (2012). Enhanced interleukin-1 activity contributes to exercise intolerance in patients with systolic heart failure. PLoS ONE.

[151] Van Tassell B.W., Arena R., Biondi-Zoccai G., Canada J., Oddi C., Abouzaki N.A., Jahangiri A., Falcao R.A., Kontos M.C., Shah K.B. (2014). Effects of interleukin-1 blockade with anakinra on aerobic exercise capacity in patients with heart failure and preserved ejection fraction (from the D-HART pilot study). Am. J. Cardiol..

[152] Ridker P.M., Thuren T., Zalewski A., Libby P. (2011). Interleukin-1β inhibition and the prevention of recurrent cardiovascular events: Rationale and design of the Canakinumab Anti-inflammatory Thrombosis Outcomes Study (CANTOS). Am. Heart J..

